# NSD2 promotes ventricular remodelling mediated by the regulation of H3K36me2

**DOI:** 10.1111/jcmm.13961

**Published:** 2018-10-18

**Authors:** Xue‐liang Zhou, Rong‐rong Zhu, Xia Wu, Hua Xu, Yun‐yun Li, Qi‐rong Xu, Sheng Liu, Huang Huang, Xinping Xu, Li Wan, Qi‐cai Wu, Ji‐chun Liu

**Affiliations:** ^1^ Department of Cardiac Surgery The First Affiliated Hospital Nanchang University Nanchang China; ^2^ Department of Obstetrics and Gynecology Jiangxi Province Hospital of Integrated Traditional Chinese and Western Medicine Nanchang China

**Keywords:** H3K36me2, myocardial infarction, NSD2, ventricular remodelling

## Abstract

Histone lysine methylation plays an important role in the regulation of ventricular remodelling. NSD2 is involved in many types of tumours through enhancing H3K36me2 expression. However, the role of NSD2 in the regulation of histone lysine methylation during ventricular remodelling remains unclear. In this study, we established cardiac hypertrophy model in C57BL/6 mice by transverse aortic constriction and found that histone lysine methylation participated in ventricular remodelling regulation via the up‐regulation of H3K27me2 and H3K36me2 expression. In addition, we constructed transgenic C57BL/6 mice with conditional knockout of NSD2 (NSD2^−/−^) in the myocardium. NSD2^−/−^ C57BL/6 mice had milder ventricular remodelling and significantly improved cardiac function compared with wild‐type mice, and the expression of H3K36me2 but not H3K27me2 was down‐regulated. In conclusion, NSD2 promotes ventricular remodelling mediated by the regulation of H3K36me2.

## 1 INTRODUCTION

In the late stage of myocardial infarction, the heart undergoes ventricular remodelling characterized by myocardial hypertrophy and interstitial fibrosis due to abnormal activation of cardiac embryonic fibroblasts.^1^ Ventricular remodelling is an independent risk factor for the development of heart failure which is a fatal disease with only 35% of patients surviving 5 years.^2^ Thus it is urgent to prevent the progression of ventricular remodelling and heart failure.^3^, ^4^

Histone lysine methylation (HKM) refers to the process of 1‐3 S‐adenosylmethionine methyl transfer to lysine residues catalysed by lysine methyltransferases and the formation of mono (me1), di (me2) or tri (me3) methylated derivatives, which can promote chromatin opening, increase DNA accessibility, initiate DNA transcription and RNA synthesis.^5^, ^6^ HKM plays an important role in the regulation of ventricular remodelling.^7^, ^8^ For example, H3K9me2 and H3K9me3 can inhibit pathological myocardial hypertrophy.^9^, ^10^ NSD2 (The Nuclear SET Domain 2) is one of the major members of the NSD family and is highly expressed in the myocardium. NSD2 is a typical histone lysine methyltransferase, which mainly catalyses the formation of H3K36me2 and promotes gene transcription.^11^, ^12^ Loss of NSD2 function would cause Wolf‐Hirschhorn syndrome characterized by cardiac malformations.^13^, ^14^ However, the role of NSD2 in ventricular remodelling has not been studied.

In this study, we found that histone methylation was involved in pathological process of ventricular remodelling in mouse models. In mice with conditional knockout of NSD2 in the myocardium, ventricular remodelling was reduced and cardiac function greatly improved compared to wild‐type mice.

## 2 MATERIALS AND METHODS

### 2.1 Antibodies

ANP and BNP antibodies were purchased from Santa Cruz Biotech (Santa Cruz, CA, USA), β‐MHC, Fibronectin, NSD2, H3K27me2, H3K36me2, H3K4me2 and H3K27me1 antibodies were purchased from Cell Signaling (Danvers, MA, USA), Collagen I, α‐SMA, Tensin, GAPDH, Histon H3 antibodies were purchased from Abcam (Cambridge, MA, USA).

### 2.2 Animal

All animals were maintained in a specific pathogen‐free facility. α‐*MHC* ^*Cre*/+^transgenic mice were purchased from Beijing Biocytogen Co. Ltd. (Beijing, China) *Nsd2* ^*fl/fl*^ C57BL/6 mice were generated by Shanghai Nanfang Research Center for Model Organisms, using conventional homologous recombination in embryonic stem cells, and the targeting strategy was shown in Figure 2A. All animal experiments were performed in accordance with the guidelines of NIH and under approved protocols of the Animal Care and Use Committee of Nanchang University.

### 2.3 Transverse aortic constriction

C57BL/6 mice and *Nsd2* ^*fl/fl*^ C57BL/6 mice were anaesthetized by intraperitoneal injection of ketamine (100 mg/kg) and xylazine (10 mg/kg), and surgery was performed using aseptic technique. Each mouse was restrained supine on a magnetic stainless steel surgical board. After skin incision, the upper half of the sternum was divided in the midline using scissors and the thymus was removed, the aortic arch was identified and freed by additional blunt dissection. A 7‐0 silk suture was placed around the aortic arch and tied tightly around a blunt needle (27 gauge). The needle was then rapidly removed. The sternotomy and the skin incision were closed with 5‐0 sutures. Sham operated animals underwent exactly the same procedure except the ligation of the aorta. The mice were kept warm on a heating pad throughout the procedure and during recovery. At 4 weeks after the surgery, cardiac function was assessed by echocardiography.

### 2.4 Histology

Isolated hearts were fixed in 10% formalin, dehydrated and paraffin embedded. The 5 μm sections were cut and stained with hematoxylin and eosin (H&E) and wheat germ agglutinin (WGA, Alexa Fluor 488 conjugate; Thermo Fisher, Rockford, IL, USA). Masson (HT15‐1KT; Sigma‐Aldrich, St. Louis, MO, USA) staining was performed to determine collagen deposition following the manufacturer's instruction. The slides were examined microscopically and the fibrotic area was determined by the area of myocardial collagen/the area of the field using Image Pro‐Plus version 6.0 (Media Cybernetics, Rockville, MD, USA) image analysis software.

### 2.5 Echocardiographic measurement

C57BL/6 mice and NSD^−/−^ C57BL/6 mice were anaesthetized by i.p. injection of pentobarbital sodium (40 mg/kg) and fixed in supine position on a heating pad. Heart rate was monitored with a standard limb lead II electrocardiogram and maintained at 50 ± 5 per minute during the echocardiography. Cardiac functions were evaluated by transthoracic echocardiography with an ultrasound machine (Visual Sonics Inc., Toronto, ON, Canada) with a 716 probe. Left ventricular systolic diameter (ESD), left ventricular diastolic diameter (EVD), ejection fraction (EF) and fractional shortening (FS) were derived automatically by the High‐Resolution Electrocardiograph System. All measurements were averaged over three consecutive cardiac cycles.

### 2.6 Preparation of nuclear extracts

The cells were incubated in 400 μL buffer A (20 mmol/L HEPES [pH 7.9], 10 mmol/L KCl, 1 mmol/L EGTA, 1 mmol/L dithiothreitol [DTT]) on ice for 15 minutes, and vortexed for 10 seconds after adding 25 μL 10% NP‐40, followed by centrifugation at 13 000 *g* for 1 minute. The pellet was incubated with buffer B (20 mmol/L HEPES [pH 7.9], 10 mmol/L KCl, 500 mmol/L NaCl, 1 mmol/L EGTA, 1 mmol/L DTT) for 15 minutes at 4°C, followed by centrifugation at 13 000 *g* for 10 minutes, and the supernatant was used as a nuclear extract.

### 2.7 Western blot analysis

Myocardial cells were lysed in cell lysis buffer (Beyotime Institute of Biotechnology, Nanjing, China) at 4°C. Protein samples were separated by 8%‐10% SDS‐PAGE, then transferred to nitrocellulose membranes (Millipore, Billerica, MA, USA) and blocked in 10% nonfat milk in TBST. Membranes were incubated with primary antibodies overnight at 4°C, followed by incubation with secondary antibodies at room temperature for 1 hour. The signals were detected using Enhanced chemiluminescence kit (Thermo Scientific, Rockford, IL, USA) and analysed by ImageQuant LAS4000 (GE, Chicago, IL, USA).

### 2.8 Statistical analysis

Data were expressed as mean ± SD and analysed by SPSS 18.0 package (SPSS Inc., Chicago, IL, USA). Comparison between two groups and among multiple groups were analysed by *t* test and one‐way ANOVA, respectively. *P* < 0.05 was considered significant.

## 3 RESULTS

### 3.1 HKM and NSD2 were involved in the pathology of ventricular remodelling

**Figure 1:**
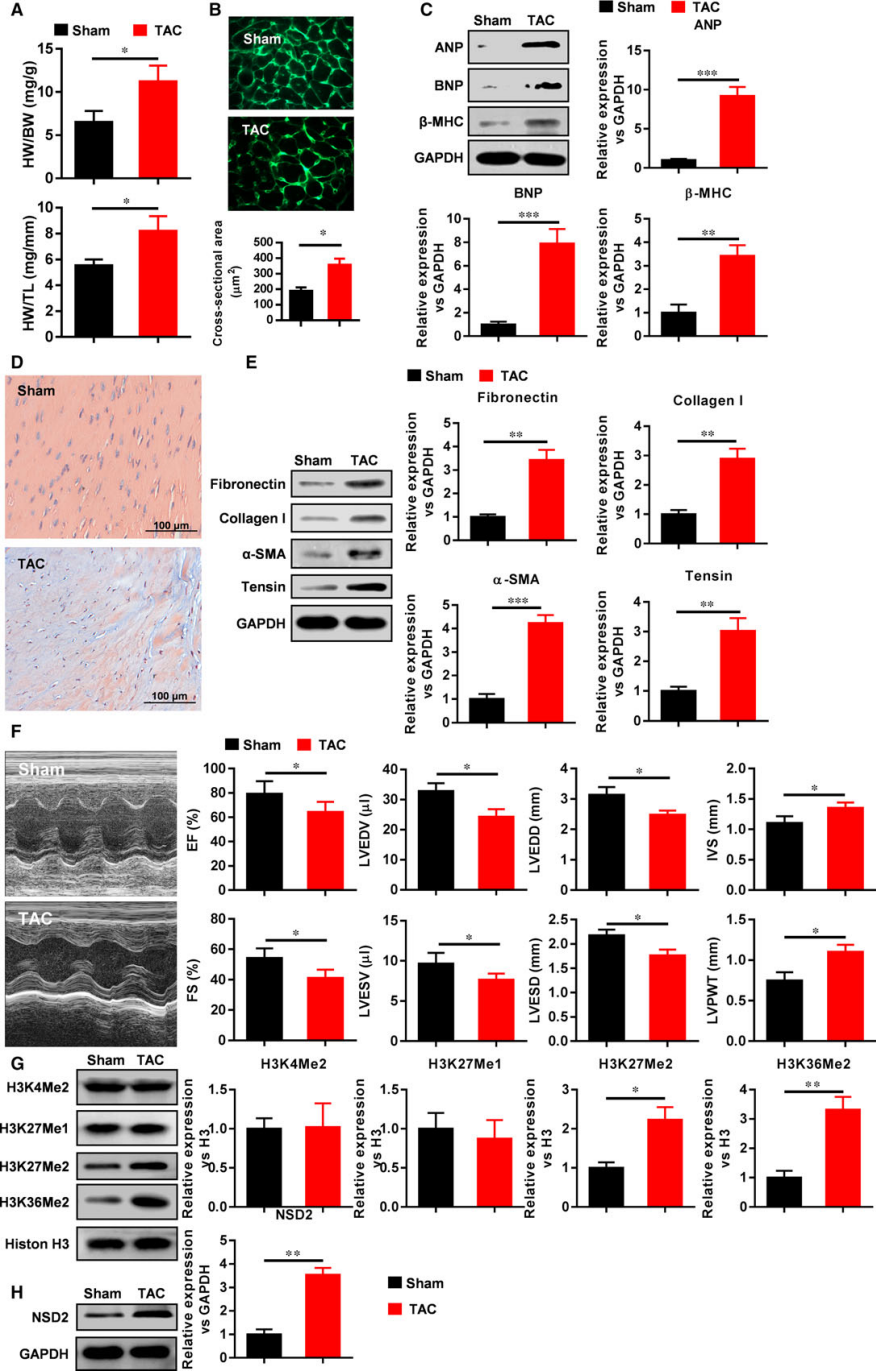
HKM was involved in the pathological regulation of ventricular remodelling mediated by NSD2. A, HW/BW and HW/TL increased after TAC; B, Myocardial cell area enlarged after TAC; C, The expression of ANP, BNP and β‐MHC increased after TAC; D, Myocardial collagen fibres increased after TAC; E, The expression of Fibronectin, Collagen I, α‐SMA and Tensin was induced by TAC; F, Cardiac function worsened after TAC; G, TAC changed the expression of H3K27me2, H3K36me2, H3K4me2 and H3K27me1; H, NSD2 expression increased after TAC (n = 6, *P < 0.05, **P < 0.01, ***P < 0.001)

Four weeks after the establishment of cardiac hypertrophy in C57BL/6 mice by transverse aortic constriction (TAC), the heart weight/body weight (HW/BW) and heart weight/tibia length (HW/TL) increased (Figure 1A), WGA staining indicated that the area of myocardial cells increased (Figure 1B), Masson's trichrome staining showed that the myocardial collagen fibres increased (Figure 1D). In addition, the expression of cardiac hypertrophy markers such as ANP, BNP, β‐MHC and myocardial fibrosis markers such as Fibronectin, Collagen I, α‐SMA, Tensin increased (Figure 1C and E). Cardiac echocardiography showed that LVPWT and IVS increased, but EF, FS, LVEDV, LVESV, LVEDD and LVESD decreased, which indicated loss of heart function in mice (Figure 1F).

Next we examined HKM status under myocardial hypertrophy and found that nuclear H3K27me2 and H3K36me2 expression increased, but nuclear H3K4me2 and H3K27me1 expression did not change (Figure 1G). Moreover, NSD2 expression was significantly up‐regulated under cardiac hypertrophy (Figure 1H). Taken together, ventricular remodelling model was successfully constructed based on the pathological aspects of cardiac hypertrophy and fibrosis, and HKM and NSD2 are involved in the pathological process of ventricular remodelling.

### 3.2 Specific knockout of cardiac NSD2 did not lead to ventricular remodeling

**Figure 2:**
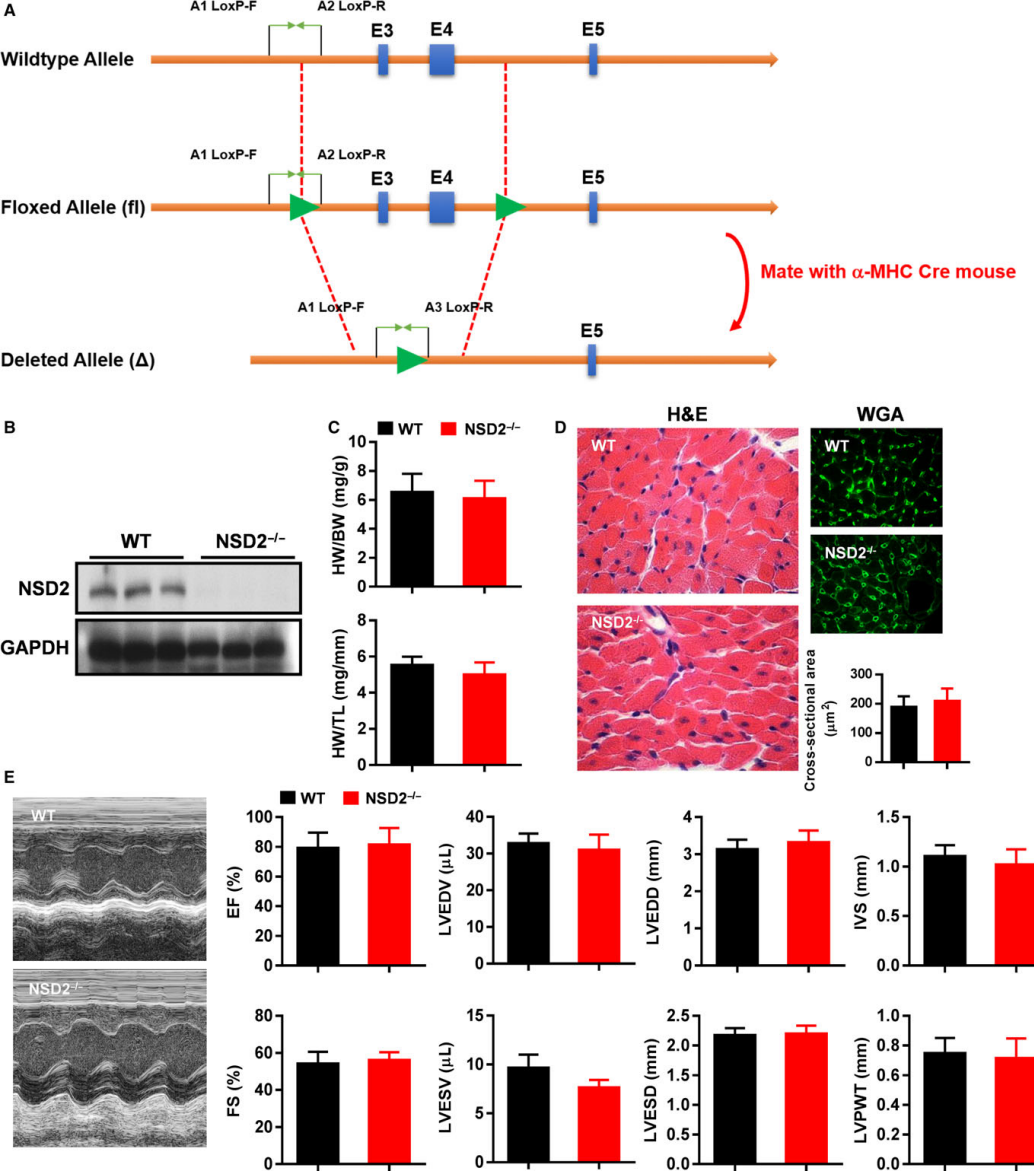
Specific knockout of NSD2 in the heart did not lead to ventricular remodelling in the mice. A, The scheme of specific knockout of NSD2 in the mice; B, NSD2 expression in NSD ^−/−^ C57BL/6 mice; C, HW/BW and HW/TL did not change in NSD ^−/−^ C57BL/6 mice; D, There was no change in myocardial cell area and collagen fibres in NSD ^−/−^ C57BL/6 mice. E, There was no change in cardiac function in NSD ^−/−^ C57BL/6 mice

To explore the role of NSD2 in ventricular remodelling, we constructed a C57BL/6 mouse with specific knockout of NSD2 in the myocardium (NSD2^−/−^ C57BL/6 mice) (Figure 2A). After confirming that NSD2 is not expressed in the myocardium of NSD2^−/−^ C57BL/6 mice, we found that there were no specific changes in HW/BW, HW/TL, myocardial cell size, myocardial cell area and cardiac function (Figure 2B‐E). These data indicate that the knockout of NSD2 does not lead to ventricular remodelling.

### 3.3 Ventricular remodelling was inhibited in NSD2^−/−^ C57BL/6 mice

**Figure 3:**
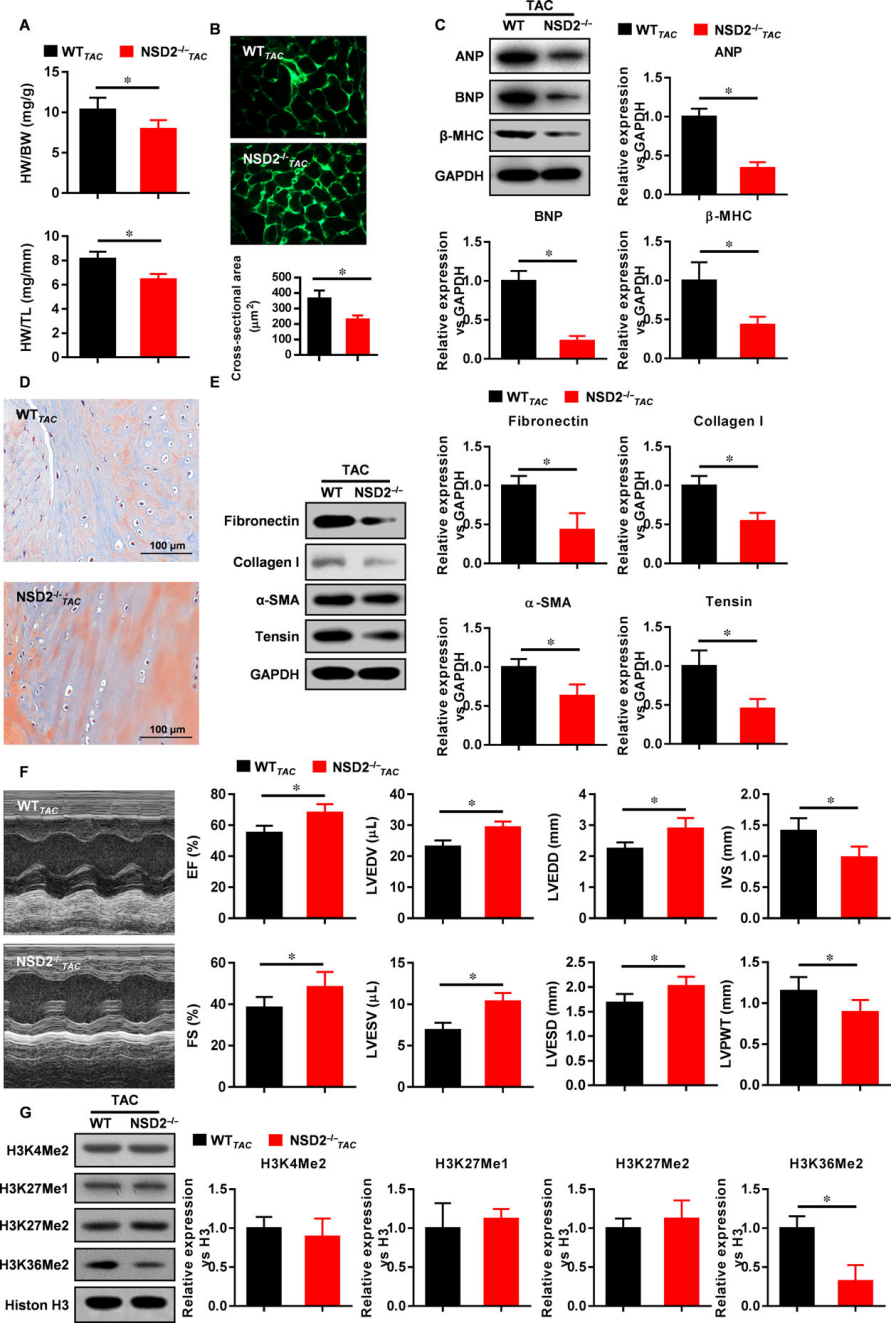
Ventricular remodelling was inhibited in NSD2^−/−^ C57BL/6 mice. A, HW/BW and HW/TL decreased after myocardial NSD2 knockout; B, Myocardial cell area decreased after myocardial NSD2 knockout; C, The expression of ANP, BNP and β‐MHC was down‐regulated after myocardial NSD2 knockout; D, Myocardial collagen fibres were decreased after myocardial NSD2 knockout; E, The expression of Fibronectin, Collagen I, α‐SMA and Tensin was down‐regulated after myocardial NSD2 knockout; F, Cardiac function was improved after myocardial NSD2 knockout; G, H3K36me2 expression was decreased after myocardial NSD2 knockout (n = 6, *P < 0.05, **P < 0.01)

HW/BW and HW/TL of NSD2^−/−^ C57BL/6 TAC mice declined compared to wild‐type (WT) mice (Figure 3A), myocardial cell area shrank, collagen fibres decreased, the expression of ANP, BNP, β‐MHC, fibronectin, Collagen I, α‐SMA and Tensin decreased, and the heart function improved (Figure 3B‐F). The expression of nuclear H3K4me2, H3K27me1, H3K27me2 did not change significantly, but nuclear H3K36me2 expression significantly decreased (Figure 3G). These results indicate that knockout of NSD2 could inhibit the process of ventricular remodelling and may be related to the down‐regulation of H3K36me2.

## 4 DISCUSSION

In this study, we found that myocardial remodelling symptoms were improved in mice with knockout of NSD2 in the myocardium, accompanied by H3K36me2 down‐regulation. Thus we speculate that NSD2‐regulated H3K36me2 is involved in the process of ventricular remodelling.

Epigenetics refers to stable and inheritable changes in gene expression without alterations in DNA sequences, and is crucially involved in gene regulation, cellular differentiation, gene imprinting, X‐chromosome inactivation and other cellular processes.^15^ As an important regulatory mechanism of epigenetics, HKM not only regulates the development and differentiation of the heart, but also participates in the occurrence and progression of ventricular remodelling.^16^, ^17^, ^18^, ^19^ MRTF‐A recruits H3K4me2/3 to ET‐1 promoter region and promotes cardiac hypertrophy and interstitial fibrosis through Angiotensin II.^20^ As a H3K9me3 demethylase, JMJD2A binds to FHL1 promoter and promotes cardiac hypertrophy.^21^ By interacting with PRC2, G9a catalyses the formation of H3K9me3, which inhibits the expression of anti‐cardiac hypertrophy genes mediated by the MEF2C complex.^10^ In this study, we found that H3K27me2 and H3K36me2 participate in the pathogenesis of ventricular remodelling which is associated with NSD2.

NSD2 is also known as MMSET/WHSC1 and contains SET domain, PHD zinc finger structure, PWWP domain and HMG box.^22^, ^23^ NSD2 mainly methylates H3K36, and is also involved in the methylation of H3K4, H3K9, H3K27, H3K79, H4K20, H4K44.^24^ NSD2‐mediated H3K36me2 promotes transcriptional activation, however, NSD2‐mediated H3K36me3 regulates RNA cleavage modification and DNA damage repair.^11^ NSD2 is known to promote tumour growth, invasion and metastasis through enhancing H3K36me2 expression, and leads to the occurrence and progression of many types of tumours including acute myeloid leukaemia, multiple myeloma, lung cancer, breast cancer and glioblastoma.^25^ Through the interaction with Sall1, Sall4 and Nanog, NSD2 regulates cardiac development and differentiation via downstream gene Nkx‐2.5 modulated by H3K36me3. However, the role of NSD2 in ventricular remodelling has not been studied. In this study we found that NSD2 expression was elevated during ventricular remodelling. The expression of H3K36me2 but not H3K27me2 was down‐regulated after specific knockout of NSD2 gene in the myocardium. In addition, knockout of NSD2 reduced the symptoms of ventricular remodelling, suggesting that NSD2 may become a new target for individualized treatment of ventricular remodelling. Writers, Readers and Erasers are the three elements of epigenetic regulation of HKM, among which Writers is a lysine methyltransferase, Readers is a lysine methylation recognition protein and Erasers is a demethylase.^7^ Further studies are needed to elucidate the molecular mechanism by which NSD2 regulates ventricular remodelling via epigenetic modifications. The activation of angiogenic key factors in NSD2‐dependent tumour progression may play the same role in myocardial growth and angiogenesis during cardiac hypertrophy.

Current understanding of NSD2 mainly focuses on its role in tumorigenesis. To our knowledge, there is still not any report on the role of NSD2 in cardiogenesis although several studies have reported on the crucial involvement of histone H3 lysine methylation in heart development.^26^, ^27^, ^28^, ^29^ This is the first report to investigate the role of NSD2 in ventricular remodelling. We found that the expression of H3K36me2 but not H3K27me2 was down‐regulated in the myocardium of NSD2 KO mice, consistent with current knowledge that NSD2 mainly methylates H3K36.^24^ Notably, a recent study showed that Nsd2‐mediated H3K36 methylation plays crucial role in adipose tissue development and function.^30^ We speculate that NSD2‐mediated H3K36me2 up‐regulation promotes transcriptional activation of key genes involved in cardiogenesis and thus contribute to ventricular remodelling. It will be important to identify these genes regulated by NSD2 which may provide new targets for the diagnosis and therapy of heart diseases.

In conclusion, HKM participates in ventricular remodelling and NSD2 promotes ventricular remodelling mediated by the regulation of H3K36me2. NSD2 is a promising target for the treatment of ventricular remodelling.
